# Modulation of auditory sensory memory by chronic clinical pain and acute experimental pain: a mismatch negativity study

**DOI:** 10.1038/s41598-018-34099-y

**Published:** 2018-10-23

**Authors:** Lu Fan, Ya-Bin Sun, Ze-Kun Sun, Ning Wang, Fei Luo, Feng Yu, Jin-Yan Wang

**Affiliations:** 1 CAS Key Laboratory of Mental Health, Institute of Psychology, Chinese Academy of Sciences, Beijing, 100101 P. R. China; 2 Department of Psychology, University of Chinese Academy of Sciences, Beijing, 100049 P. R. China; 3 Department of Neurosurgery, Chinese PLA General Hospital of Jinan Military Command, Jinan, 250031 P. R. China

## Abstract

Pain, especially chronic pain, can lead to cognitive deficits. Mismatch negativity (MMN) is a change-specific component of the auditory event-related brain potential (ERP) that is thought to provide a unique window into sensory memory processes. The present study was designed to determine how chronic and acute pain affects auditory sensory memory. In experiment 1, MMNs elicited by standard and deviant auditory stimuli at short and long inter-stimulus intervals (ISIs) were compared between trigeminal neuralgia (TN) patients and demographically matched healthy controls (HCs). The TN patients were found to have stronger attenuation of the MMN at longer ISIs than HCs. Correlation analysis revealed a significant positive correlation between the sensory subscale of McGill Pain Questionnaire and MMN amplitude reduction across ISI conditions. In experiment 2, MMNs recorded before, during, and after the cold pressor test were compared in healthy subjects. MMN amplitude was significantly reduced during pain exposure and recovered immediately thereafter. These results suggest that both chronic pain and acute pain can interfere with automatic change detection processes in the brain. This study provides the first evidence that chronic pain patients have a faster auditory memory trace decay than HCs.

## Introduction

Pain is a subjective experience that involves interactions among sensory, affective, and cognitive factors^1,2^. When pain is chronic, it can produce sequela beyond the sensation of pain itself^3–5^. Indeed, substantial evidence indicates that chronic pain is associated with a general decline in cognitive functions, including attention, memory, and executive function^6,7^.

The division of resources theory holds that pain-related cognitive impairment may be a consequence of limited resources being consumed by pain processing and management^3–5^. According to this theory, competition between pain and cognition for processing resources may impair cognitive abilities in chronic pain patients. In a functional magnetic resonance imaging (fMRI) study of attention-related cerebral responses, Martinsen *et al*. found that, relative to healthy controls (HCs), chronic pain patients had reduced activation in brain regions implicated in cognitive processing^8^. In another fMRI study, Aizawa *et al*. found that patients with irritable bowel syndrome had cognitive flexibility impairments that were associated with altered activity in the dorsolateral prefrontal cortex and hippocampus^9^.

Electroencephalography (EEG) studies of pain effects on event-related potentials (ERPs) have also provided evidence of pain-associated cognitive impairment. The mismatch negativity (MMN) has been demonstrated to be affected by ongoing pain^10–13^. Experimentally, the MMN is elicited reliably by a detectable auditory change or regularity violation in a sequence of frequent standardized stimuli^14^. It is thought to arise from an automatic comparison between ongoing sensory input and the memory trace of the previous stimulus, and thus to reflect some aspect of sensory memory processing^15^. Two major generator sources of MMN in the brain have been proposed, one bilaterally generated by the auditory cortices (temporal component) and the other by the frontal cortex (frontal component)^14,16^. The temporal and frontal component has been suggested to reflect pre-perceptual change detection and involuntary attention switch to auditory change respectively^17,18^.

Dick *et al*. provided the first evidence that the MMN was affected by pain when they demonstrated that MMN amplitude was increased in pain patients following analgesic treatment^12^. Conversely, the same group did not find evidence of the MMN being affected by experimentally induced ischemic pain^11^. In a recent study, Yao *et al*. reported unchanged MMN amplitudes but delayed MMN latency in low back pain and neck pain patients^13^. Most recently, Choi *et al*. showed that the amplitude of MMN’s magnetoencephalographic equivalent was smaller in patients with fibromyalgia than in healthy subjects^10^.

To date, the effect of chronic pain on sensory memory, as reflected by the MNN response to ISI lengthening, has not been examined and the effect of experimentally induced pain on MMN has not been reexamined since Dick *et al*.’s 2006 study. The aim of the present study was to examine how both chronic and acute pain affect auditory sensory memory. MMNs elicited by auditory stimuli at short and long ISIs were compared between chronic pain patients and HCs. We hypothesized that MMN attenuation in long ISI trials would be stronger in chronic pain patients than in HCs. In addition, MMNs recorded before, during, and after acute pain in the cold pressor test (CPT) were compared in healthy subjects. We predicted that the MMN amplitude would be reduced by acute cold pain and would recover after the pain subsided.

## Methods

### Participants and design

A total of 71 individuals participated in this study, which consisted of experiments 1 and 2. Experiment 1 was designed to investigate the effects of chronic pain on the maintenance of the sensory memory trace, indexed by the MMN. It had a between-subject design with a group of 26 chronic pain patients suffering from trigeminal neuralgia (TN) and a group of 25 age and gender matched HCs. Experiment 2 was designed to examine the modulation of MMN by experimentally induced acute pain. It employed a within-subject design with a group of 20 healthy, undergraduate and graduate students who underwent the cold pressor test (CPT).

The TN patients were recruited from the Department of Neurosurgery, Chinese PLA General Hospital of Jinan Military Command, which receives TN patients from all over the country for microvascular decompression surgery. TN diagnosis was confirmed by a neurosurgeon (author F. Y.) based on inquiry, physical examination, and magnetic resonance imaging. Inclusion criteria for the TN patients were: (a) idiopathic TN, as defined by the International Headache Society^19^; (b) persistent chronic pain for at least 6 months; and (c) normal hearing and normal or corrected to normal vision. Patients were excluded if they had: (a) other types of acute or chronic pain; (b) a history of any other medical, neurological, or psychiatric disorder; or (c) current alcohol or substance abuse problems. All TN patients suffered from unilateral (13 left and 13 right) facial pain. The mean duration of pain history was 5.48 ± 5.59 years. The experiments were performed on the day before neurosurgical interventions (i.e., microvascular decompression). During the experiment, pain was controlled with carbamazepine. Based on the present pain intensity scale of the McGill Pain Questionnaire (MPQ), all patients rated their current pain less than 3 (mean 1.59 ± 1.37; range, 0–5).

The HCs in Experiment 1 were recruited through local advertisement posted in the local community. The HC group were demographically matched to the TN group. The exclusion criteria applied to the TN group were also applied to the HC group. Two patients who refused to undergo EEG were excluded from the study. Another 4 TN patients and 3 HC subjects were excluded because they could not understand the instructions. Additionally, 3 TN patients and 4 HCs were excluded from the analyses due to excessive EEG artifacts. The demographic information of the Experiment 1 participants is summarized in Table 1.

**Table 1:** Demographic information and neuropsychological measurement scores in experiment 1.

Variable	TN (n = 17)		HC (n = 18)	
	Mean	SD	Mean	SD
Age (years)	48.94	13.63	46.06	5.43
Education (levels)^a^	2.29	1.21	2.11	0.83
Fear of Pain Questionnaire (FPQ) (30–150)	63.71	19.78	64.17	22.18
**Pain Anxiety Symptoms Scale (PASS-20)**				
Total (0–100)	41.53**	16.99	19.61	20.19
Cognitive anxiety (0–25)	13.29**	5.27	5.39	5.39
Physiological anxiety (0–25)	5.53	5.55	4.83	6.08
Fear (0–25)	5.76	4.97	3.11	4.17
Avoidance (0–25)	16.94**	6.29	6.28	6.83
**Depression Anxiety and Stress Scale (DASS-21)**				
Total (0–63)	14.12	10.73	8.83	7.29
Depression (0–21)	4.59	3.59	2.06	2.26
Anxiety (0–21)	2.88	3.28	2.72	2.59
Stress (0–21)	6.65	5.79	4.06	3.28
**McGill Pain Questionnaire (SF-MPQ)**				
Total (0–45)	16.76	6.18		
Sensory index (0–33)	12.75	5.32		
Affective index (0–12)	4.01	2.11		
	**n**	**%**	**n**	**%**
**Gender**				
Male	11	64.71	13	72.22
Female	6	35.29	5	27.78

^a^The level of education is measured by the grading method, 1 represents the lowest degree (primary school), 7 represents the highest degree (doctor degree).

**P < 0.01 for TN vs. HC.

TN, Trigeminal neuralgia patients; HC, healthy controls.

The participants in Experiment 2 (10 males; mean age 23.50 ± 3.83 years) were recruited through advertisements posted on local college campuses. The exclusion criteria were: (a) a history of cardiovascular disease, (b) a history of fainting or seizures, (c) a history of Reynaud’s syndrome, or (d) a recent injury or open cut affecting the hand^20^. Two participants could not endure the cold pressor pain and quit the study, and another 2 were excluded due to excessive EEG artifacts. Thus, the final sample size in Experiment 2 was 16 (9 males; mean age, 23.44 ± 3.33 years).

All study participants gave their written informed consent after they had received a detailed explanation of the experimental protocol. They were informed that they could terminate the experiment at any time. The study was performed in accordance with relevant guidelines and regulations. Ethical approval was granted by the Institutional Review Board of the Institute of Psychology, Chinese Academy of Sciences.

### Experiment 1

#### Questionnaires

Questionnaires were used to collect information on demographics, medical history, and emotional status. All participants were asked to fill out Chinese versions of the Fear of Pain Questionnaire (FPQ), the 20-item Pain Anxiety Symptoms Scale (PASS-20), and the 21-item Depression Anxiety and Stress Scale (DASS-21). TN patients also completed the short form (SF)-MPQ, to provide a quantitative evaluation of the sensory and affective aspects of their pain. If the participant could not read, the experimenter read the scales to them, and the participant reported their answers verbally.

The FPQ is a 30-item survey that has been widely used as a self-reported measure of pain-related fear in (chronic) pain syndromes as well as in non-clinical samples. For each painful situation described on the FPQ, respondents rate how fearful they are on a scale from 1 (not at all) to 5 (extreme)^21^. This scale has a good reported internal consistency, with Cronbach’s α of 0.93. The Chinese FPQ (edition III) used in the experiment was translated and validated by a research group at Southwest University, Chongqing, China^22^.

The PASS-20 consists of four 5-item subscales (cognitive, escape/avoidance, fear, and physical anxiety) and is intended to measure pain-related anxiety and fear responses^23^. All items are rated on a frequency scale from 0 (never) to 5 (always). The PASS-20, a short form version of the original 40-item PASS, has been demonstrated to have good internal consistency (mean α = 0.81) and to correlate strongly (mean r = 0.95) with the original scales^23^. The four subscales and full scale of the Chinese PASS-20, used in this study, have been shown to have good internal consistency with a Cronbach’s α of 0.72–0.92^24^.

The DASS-21 is a self-reported questionnaire that is widely used to assess depression, anxiety, and stress dimensions in clinical and nonclinical groups^25^. Each of its 21 items are rated on a 4-point (0–3) scale of frequency or severity of respondent experiences over the last week. Respondents rated how much each item applied to them (0 = does not apply to me at all, 3 = applies to me very much). This scale has high reliability, with Henry reporting a Cronbach’s α of 0.93^26^. The Chinese version of DASS-21 has internal consistency indices (Cronbach’sα values) of 0.83, 0.80, and 0.82 for its Depression, Anxiety, and Stress subscales, respectively, and 0.92 for the total scale^24^.

Pain intensity was assessed with the SF-MPQ, which consists of 15 pain descriptors (11 sensory and 4 affective) rated on a 4-point scale (0 = none, 1 = mild, 2 = moderate, 3 = severe)^27^. The SF-MPQ provides information on the sensory, affective, and overall intensity of pain with a sensory index, affective index, and pain rating index, respectively. It includes the present pain intensity index of the standard MPQ and a visual analogue scale. The sensory and affective dimensions of the SF-MPQ were shown to have good internal consistency (Cronbach’sα valuesof 0.78 and 0.76, respectively), and were translated into a Chinese version and standardized^28^.

#### Stimuli and procedure

Auditory stimuli consisted of standard tone (1000 Hz) and occasional deviant tone (2000 Hz). Both were pure sine wave tones with an intensity of 80 dB SPL (sound pressure level) and a duration of 50 ms with 5-ms rise and fall times. Stimuli were presented in two blocks, each having a constant ISI of 500 ms (short) or 2500 ms (long). Each block contained 500 auditory stimuli and the order of blocks (short-ISI and long-ISI) was counterbalanced among participants. The standard and deviant tone occurrence probabilities were fixed at 90% and 10% for both ISI conditions, resulting in 450 standard and 50 deviant tones in each block. Within each block, stimuli were presented pseudo-randomly with the constraints of at least three standard tones being displayed at the beginning of the block and that deviant tones were separated by at least two standard tones^29^.

Participants were seated in a comfortable armchair in a dim, electrically shielded room. The stimuli were generated by a PC running E-prime software (Psychology Software Tools, Inc., Sharpsburg, PA) and were delivered binaurally through earphones. During EEG recordings, participants watched a silent documentary movie (*Winged Migration*) while listening passively to auditory stimuli via earphones. Subjects were instructed to pay attention to the video clips and to ignore the tones. They were told that they would be quizzed on content of the movie. During EEG recordings, participants were asked to refrain from frequent blinking and head movements. Between blocks, they were allowed to take a short break. On average, the task lasted for a total of about 30 min.

#### EEG recording and data preprocessing

EEG data were recorded with a 64-channel Biosemi Active Two system (BioSemi, Amsterdam, The Netherlands) with electrodes positioned according to the extended 10–20 system. Signals were referenced online to the common mode sense-driven right leg ground and digitized at 512 Hz with 0.01-Hz high-pass and 100-Hz low-pass filtering. The impedance of each electrode was <5 kΩ. Vertical eye movements were monitored with bipolar recordings above and below the left eye. Horizontal eye movements were monitored with electrodes placed at the outer canthus of each eye. Two additional EEG signals were recorded from the bilateral mastoids, M1 and M2, for computing temporal MMN, and another electrode was placed at the tip of the nose for off-line referencing.

MATLAB (version 8.3., The MathWorks Inc., Natick, MA) and EEGLAB toolbox^30^ were used for the offline analysis of EEG data. All data were first re-referenced to nose tip and then re-referenced to M1 and M2. They were all band-pass filtered in the range of 1–25 Hz. Trial epochs starting 100 ms before and ending 400 ms after the onset of auditory stimulus were extracted and then baseline corrected. Artifacts related to eye movements and cardiac responses were removed by independent component analysis^31^. Trials with EEG amplitudes larger than 100 μV were excluded from further analysis. At least 70% artifact-free standard and deviant trials were preserved for each ISI condition (short and long). The remaining trial epochs were averaged separately to compute standard and deviant ERPs under each experimental condition.

To obtain the MMN, a difference wave was computed by subtracting the individual grand average responses to standard stimuli from those to deviant stimuli for each ISI condition^32^. Based on visual inspection of the difference waveforms, as well as previous studies^12,33,34^, the MMN component was measured as the mean amplitude of the difference wave within the 150–200 ms post-stimulus time window. The MMN was recognized as a characteristic negative deflection over the frontal and central scalp sites and as positive wave over the posterior temporal regions^35,36^, and was thus analyzed within frontocentral (F3, Fz, F4, C3, Cz, C4) and temporal (M1, M2) regions of interest (ROIs). For mastoid re-referenced data, the MMN was analyzed only within the frontocentral region. To assess the responses elicited by standard and deviant tones *per se*, peak amplitude and peak latency of the N1 (50–175 ms) and P2 (120–280 ms) components were measured^33^.

#### Statistical analysis

Demographics and pain-related neuropsychological assessment results were compared between patient and control groups with Student’s *t*-tests and chi-squared analyses where appropriate. The N1 and P2 components elicited by auditory stimuli at Cz were analyzed with three-way group (TN vs. HC) × stimulus type (standard vs. deviant) × ISI (short vs. long) repeated measures (rm) analyses of variance (ANOVA) of peak amplitude and peak latency. For the nose-referenced MMN, three-way group × ISI × hemisphere rmANOVAs were conducted separately at frontocentral sites and temporal sites. To better elucidate the ISI effects, separate two-way group × ISI ANOVAs, split by hemisphere, were performed. Post-hoc pairwise comparisons with Bonferroni corrections were carried out as follow-up analyses of ANOVA revealed significant interactions. The Greenhouse-Geisser correction was applied to control for non-sphericity where appropriate. Corrected probability values are reported. The same analyses were conducted on mastoid-referenced MMN components recorded at frontocentral sites. To examine whether the electrophysiological activities were associated with pain perception, Pearson correlation analyses were performed between MMN amplitude (at each ROI for each ISI condition) and MPQ scores (including subscale scores). All statistical analyses were conducted in SPSS version 19.0 (SPSS Inc., Chicago, IL). The significance level was set at *P* < 0.05.

### Experiment 2

#### Stimuli and procedure

The CPT was conducted in a custom-made plastic tank (25 × 25 × 16 cm^3^) holding ice and water. A thermostat-controlled electric pump was used to propel water flow, while maintaining temperature 4 ± 1 °C. Ice was packed into a mesh pocket submersed in the water to prevent direct contact between the ice and the participant’s skin. A similar tank of warm water (32–34 °C) was used for hand immersion before the CPT task, to reduce inter-subject differences in hand temperature.

The physical parameters of the auditory stimuli were the same as in Experiment 1. Standard and deviant auditory stimuli were presented pseudo-randomly across three blocks (i.e., CPT baseline, immersion, recovery; details below) with a 400 ms ISI. Each block contained 500 auditory stimuli (400 standard and 100 deviant), and lasted approximately 4 min.

The CPT commenced with each participant placing his or her nondominant hand in the warm water for 4 min (baseline phase), during which auditory stimuli were delivered binaurally through earphones and EEG signals were recorded. Then, the participant transferred the immersed hand from the warm water immediately to the cold water, where it remained immersed for 4 min (maximum tolerance time) or until the participant felt too uncomfortable to continue (immersion phase). Subsequently, the participant’s hand was exposed to the room air (26 °C) for 4 min (recovery phase).

During the immersion and recovery phases, participants reported their perceived pain intensity and unpleasantness on 0–9 scales at 15 s, 30 s, 60 s, 120 s, 180 s, 240 s, 300 s, 360 s, 420 s, and 480 s after the beginning of the immersion. Auditory stimuli were delivered and EEG signals were recorded throughout the experiment.

#### EEG recording and data analysis

EEG recording and data processing were performed as in Experiment 1 except that the ERPs were analyzed with nose-referenced data only. The self-reported pain intensity and unpleasantness ratings were analyzed with one-way rmANOVAs followed by Dunnett’s tests.

Peak amplitudes and peak latencies of auditory stimulus elicited N1 and P2 components recorded at Cz were analyzed with two-way condition (baseline, immersion, recovery) × stimulus type (standard vs. deviant) rmANOVAs. The MMN datasets at frontocentral (F3, Fz, F4, C3, Cz, C4) and at temporal (M1, M2) sites were each subjected to two-way condition × hemisphere rmANOVAs. Condition effects were detected with one-way ANOVAs followed by Dunnett’s tests. Analyses of the correlations between MMN amplitude and self-reported ratings were also conducted. All statistical analyses were conducted in SPSS version 19.0 (SPSS Inc., Chicago, IL). The significance level was set at *P* < 0.05.

## Results

### Chronic clinical pain

#### Demographics and neuropsychological measures

The groups’ demographic characteristics and neuropsychological findings are presented in Table 1. The TN patient and HC groups were well matched for age (*t* = 0.83, *P* = 0.411), gender (*χ*^2^ = 0.23, *P* = 0.725), and education level (*t* = 0.52, *P* = 0.609). The TN patients exhibited a higher level of pain-related anxiety than HCs, manifested as more anxiety thoughts (PASS cognitive subscale, 13.29 ± 5.27 vs. 5.39 ± 5.39, *t* = 4.39, *P* < 0.001) and increased avoidance behaviors (PASS avoidance subscale, 16.94 ± 6.29 vs. 6.28 ± 6.83, *t* = 4.81, *P* < 0.001) when experiencing suffering. No other differences were observed between the two groups.

#### ERPs elicited by standard and deviant tones

**Figure 1:**
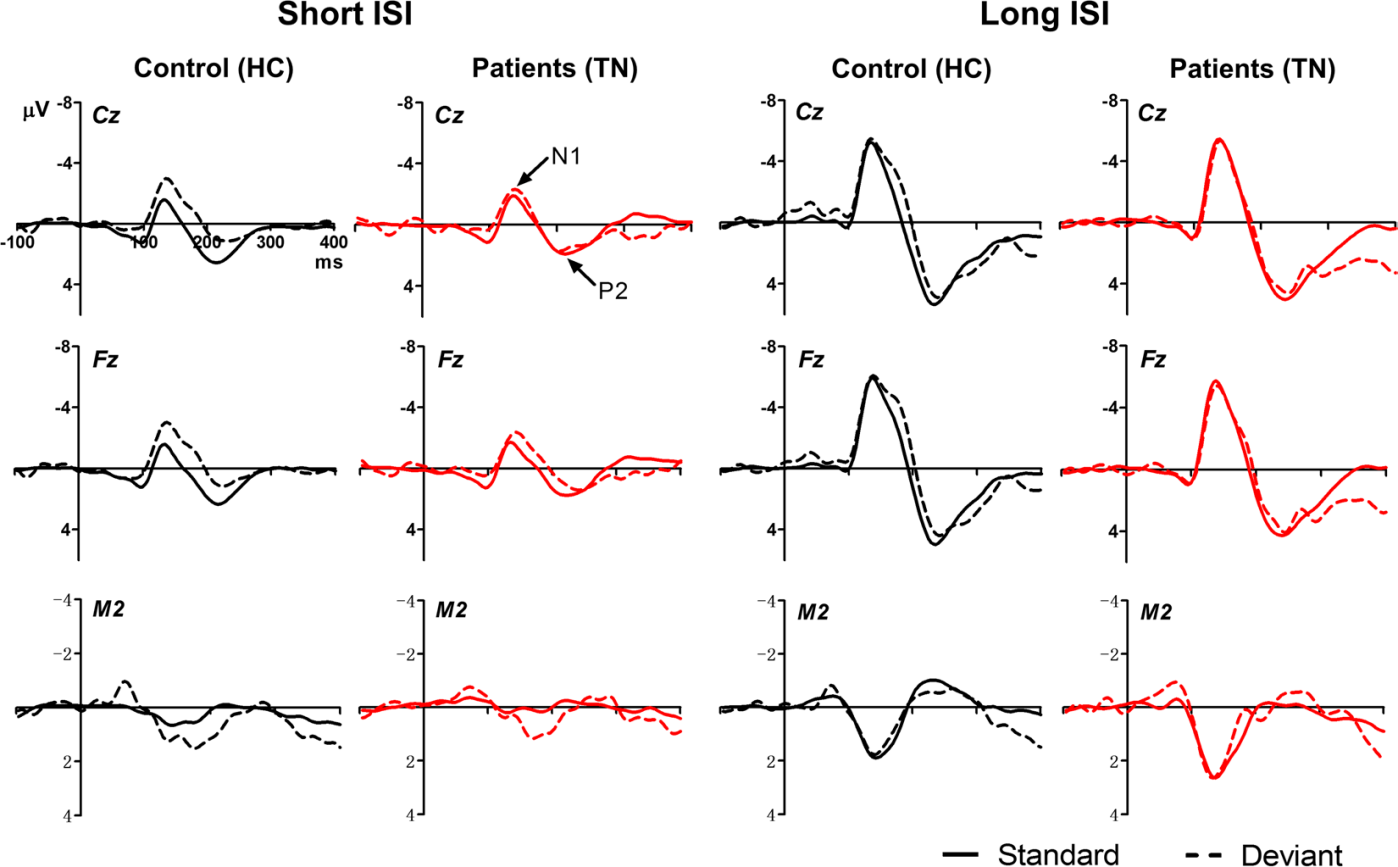
ERPs produced in response to standard and deviant tones in Experiment 1. Grand average waveforms for TN patients (red) and HCs (black) are shown for Cz, Fz, and M2 electrodes under short- (left panel) and long-ISI (right panel) conditions.

Grand average ERPs elicited by auditory stimuli with respect to stimulus type (standard vs. deviant), experimental group (TN vs. HC), ISI condition (short vs. long), and electrode site (Cz, Fz, and right mastoid) are shown in Fig. 1. The peak amplitudes and latencies of the N1 and P2 components at Cz are presented in Table 2. We observed main effects of ISI on N1 (*F*_1,33_ = 33.16, *P* < 0.001, *ηp*^2^ = 0.50) and P2 *(F*_1,33_ = 60.69, *P* < 0.001, *ηp*^2^ = 0.65), with larger amplitudes being recorded in the long-ISI condition than in the short-ISI condition. There was also a main effect of stimulus type on N1 amplitude (*F*_1,33_ = 21.27, *P* < 0.001, *ηp*^2^ = 0.39), with N1 being larger with deviant stimuli than with standard stimuli.

**Table 2:** Peak amplitude and latency of N1 and P2 to standard and deviant tones under different ISI conditions at Cz electrode in experiment 1.

	Amplitude (μV)		Latency (ms)	
	TN	HC	TN	HC
***Standard N1***				
500-ms ISI	−2.05 (1.72)	−1.81 (1.56)	136.90 (6.17)	125.26 (28.42)
2500-ms ISI	−5.77 (3.19)	−5.63 (2.11)	139.66 (9.66)	132.42 (19.20)
***Standard P2***				
500-ms ISI	2.34 (1.48)	2.96 (1.41)	210.43 (31.59)	212.07 (30.76)
2500-ms ISI	5.60 (2.23)	6.08 (1.69)	241.45 (17.20)	238.98 (21.25)
***Deviant N1***				
500-ms ISI	−3.00 (2.43)	−3.70 (1.71)	134.38 (24.89)	129.38 (21.64)
2500-ms ISI	−6.15 (3.71)	−6.62 (2.19)	137.13 (25.42)	148.48 (19.00)
***Deviant P2***				
500-ms ISI	3.36 (2.04)	2.77 (2.31)	229.27 (31.91)	242.45 (26.80)
2500-ms ISI	5.91 (3.18)	5.83 (3.34)	243.75 (20.16)	242.23 (18.91)

TN, Trigeminal neuralgia patients; HC, healthy controls.

Additionally, we found main effects of ISI on N1 (*F*_1,33_ = 6.98, *P* = 0.013, *ηp*^2^ = 0.18) and P2 (*F*_1,33_ = 19.97, *P* < 0.001, *ηp*^2^ = 0.38) latencies, where the peak latencies in the long-ISI condition were delayed compared to those in the short-ISI condition. There was also a main effect of stimulus type on P2 latency (*F*_1,33_ = 10.45, *P* = 0.003, *ηp*^2^ = 0.24) and a stimulus type × ISI interaction (*F*_1,33_ 6.98, *P* = 0.012, *ηp*^2^ = 0.18), with the peak P2 latency in response to standard stimuli being faster than that in response to deviant ones in the short-ISI condition, and no stimulus difference in the long-ISI condition (Table 2). There were no other significant group effects or interactions.

#### MMN

**Figure 2:**
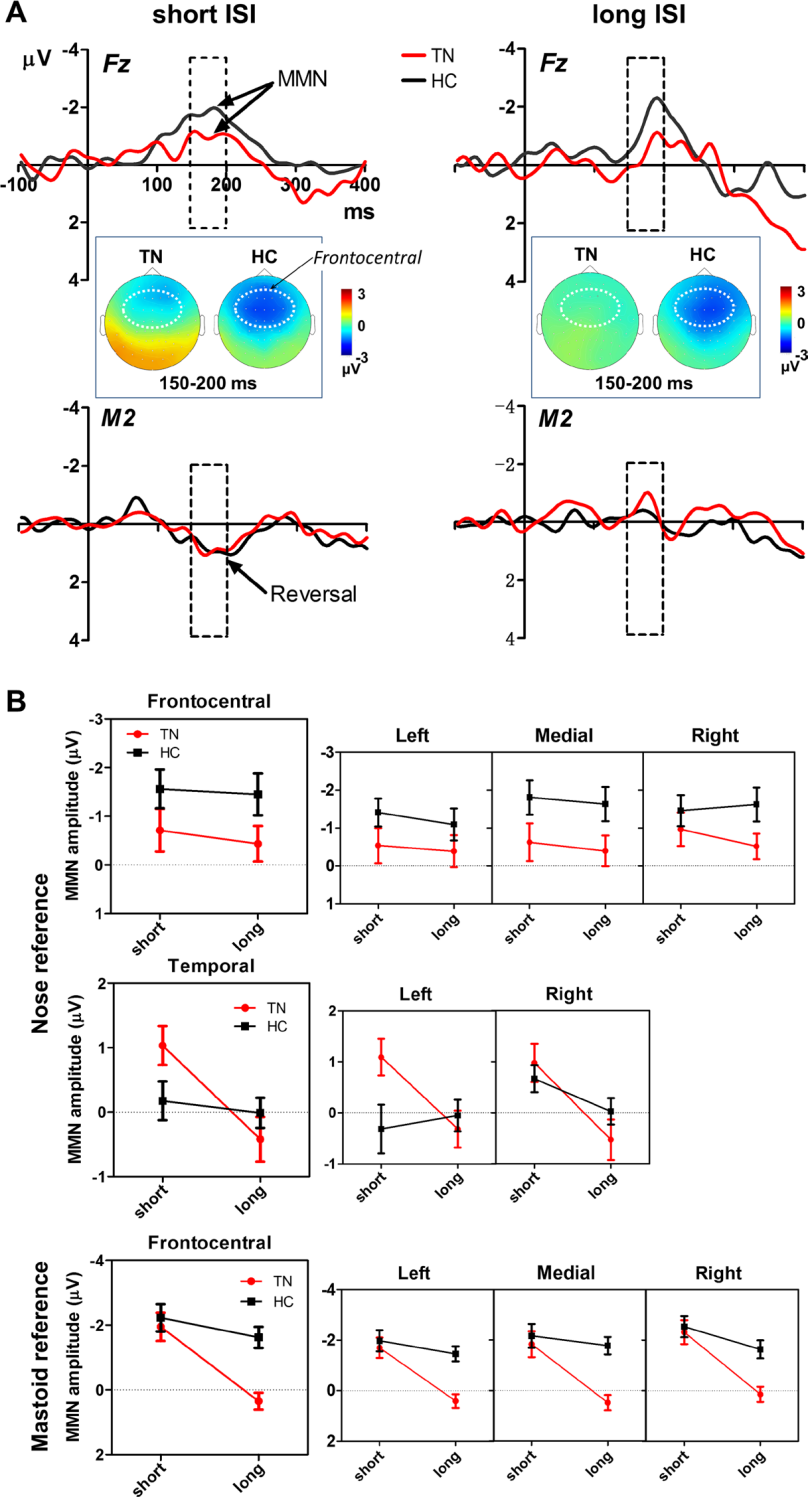
Comparison of MMN amplitudes between TN patients and HCs in different ISI conditions. (**A**) MMN grand average waveforms at frontal and temporal regions shown with scalp topographical maps. The dashed boxes superimposed on waveforms mark the MMN time window (150–200 ms). Scalp topographies of MMN amplitude are shown in this time window, with a white dotted oval indicating the frontocentral ROI. Note the typical negative deflection at Fz and a polarity reversal (positive deflection) at the mastoid. (**B**) Nose-referenced and mastoid re-referenced MMN data at frontocentral and temporal regions. Reduction of MMN amplitude with ISI prolongation was more pronounced in the TN group than in the HC group. Error bars indicate standard errors.

The difference waveforms (deviant minus standard) that constitute the MMN in short- and long-ISI conditions are presented in Fig. 2. The MMN was evident for both groups. In the short-ISI condition, a typical negative deflection at Fz and a polarity reversal (positive deflection) at the mastoid were seen for both groups. In the long-ISI condition, there was a marked negativity at Fz for both groups, but not a strong polarity reversal at the mastoid.

#### Frontocentral MMN

There was a main effect of group (nose reference) on mean MMN amplitude (Fig. 2B) at frontocentral sites, with TN patients having smaller amplitudes than the control group (*F*_1,33_ = 5.43, *P* = 0.026, *ηp*^2^ = 0.14), as well as a main effect of hemisphere, with higher MMN amplitudes being recorded from the right hemisphere (F4, C4) than from the left hemisphere (F3, C3) (*F*_2,66_ = 3.56, *P* = 0.034, *ηp*^2^ = 0.10). No ISI effects on frontocentral MMNs were found. Group × ISI two-way ANOVAs for each hemisphere revealed main effects of group on the MMN at medial sites (Fz, Cz) (*F*_1,33_ = 7.13, *P* = 0.012, *ηp*^2^ = 0.18) and at right hemisphere sites (*F*_1,33_ = 4.21, *P* = 0.048, *ηp*^2^ = 0.11), but not at left hemisphere sites.

Similarly, three-way ANOVAs of the mastoid-referenced data (Fig. 2B) revealed main effects of group (*F*_1,33_ = 8.59, *P* = 0.006, *ηp*^2^ = 0.21) and hemisphere (*F*_2,66_ = 7.27, *P* = 0.005, *ηp*^2^ = 0.18) on MMN amplitude. Unlike the nose-referenced data, there was a significant effect of ISI on MMN amplitude (*F*_1,33_ = 16.94, *P* < 0.001, *ηp*^2^ = 0.34) and a significant group × ISI interaction (*F*_1,33_ = 5.78, *P* = 0.022, *ηp*^2^ = 0.15). Subsequent post-hoc analysis indicated that the TN patients had a smaller MMN than the HC group in the long-ISI (*P* < 0.001), but not the short-ISI (*P* = 0.643), condition. Within-group contrasts showed a marked reduction in MMN amplitude for TN patients from the short to the long ISI (*P* < 0.001), whereas the MMN amplitude remained relatively stable across ISIs in the HC group (*P* = 0.258).

#### Temporal MMN

A three-way group × ISI × hemisphere ANOVA at temporal sites (left and right mastoids) revealed a main effect of ISI (*F*_1,33_ = 8.80, *P* = 0.006, *ηp*^2^ = 0.21), indicating that temporal MMN amplitude decreased with ISI prolongation. This decrease was more pronounced in the TN patient group than in the HC group, yielding an ISI × group interaction (*F*_1,33_ = 5.27, *P* = 0.028, *ηp*^2^ = 0.14) (Fig. 2B). A Bonferroni post-hoc analysis confirmed that the TN patients had a significant reduction in MMN amplitude from the short to the long ISI (*P* = 0.001), whereas MMN amplitude did not differ significantly between the two ISI conditions in the HC group (*P* = 0.634). In addition, the post-hoc comparisons showed a marginally significant difference between TN vs. HC for the short ISI (*P* = 0.053). No other significant differences were found.

#### Correlations

**Figure 3:**
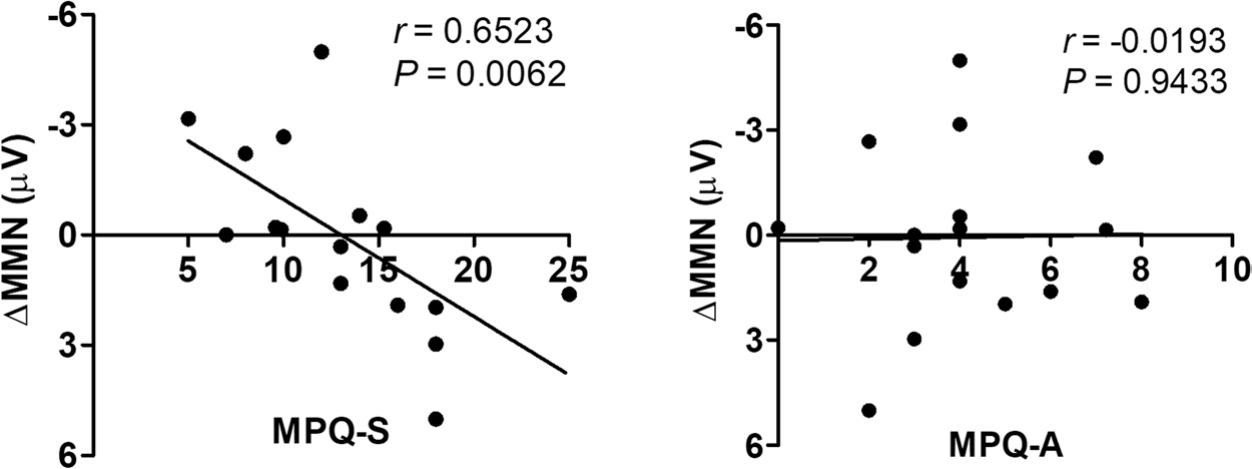
Correlation between MPQ subscale scores and MMN amplitude reduction across ISI conditions in TN patients. The sensory (left panel), but not the affective (right panel), subscale of the MPQ correlated with MMN amplitude reduction.

There was a significant correlation between the sensory, but not the affective, MPQ subscale and MMN amplitude reduction across ISI conditions (long minus short) (*r* = 0.6523, *P* = 0.0062) (Fig. 3), indicating that a more intense pain experience results in a more rapid MMN amplitude attenuation with increasing ISI.

### Acute experimental pain

#### CPT

**Figure 4:**
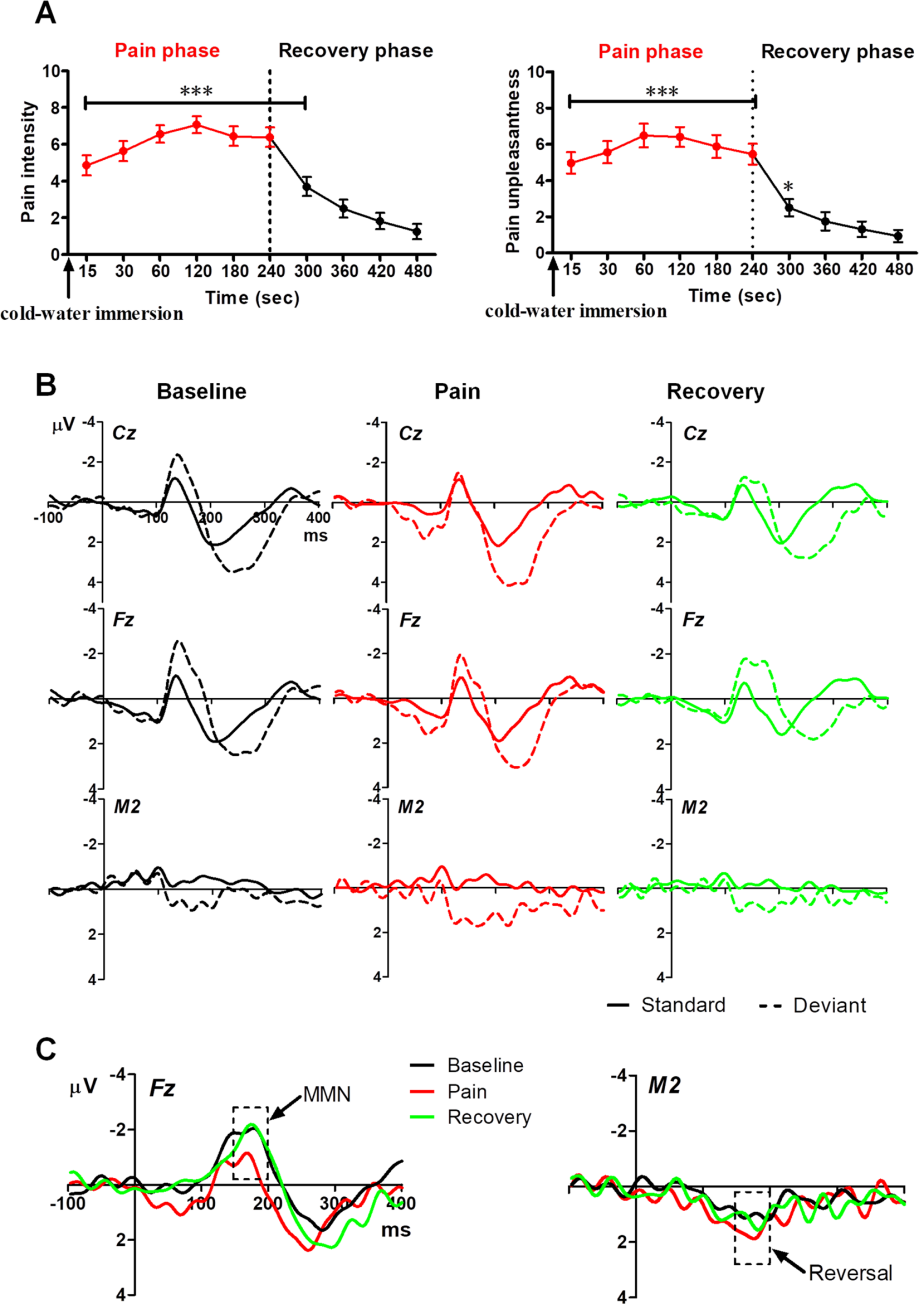
ERPs produced in response to standard and deviant tones and MMN grand average waveforms in Experiment 2. (**A**) Ratings of pain intensity and unpleasantness in cold-induced pain (red) and recovery (black) phases. *P < *0*.*05*, ***P < *0*.*001*, compared to ratings at 480 s. (**B**) Grand average waveforms generated in response to standard and deviant tones in the baseline (black), cold pain (red), and recovery (green) periods. (**C**) MMN grand average waveforms in different experimental phases. The dashed boxes superimposed on the waveforms mark the time window of the MMN (150–200 ms).

Changes in perceived pain intensity and unpleasantness ratings over time during (15 s, 30 s, 60 s, 120 s, 180 s, 240 s) and after ice-water immersion (300 s, 360 s, 420 s, 480 s) are illustrated in Fig. 4A. One-way rmANOVAs revealed revealed significant main effects of time on pain intensity (*F*_9,135_ = 36.11, *P* < 0.001, *ηp*^2^ = 0.71) and pain unpleasantness (*F*_9,135_ = 35.93, *P* < 0.001, *ηp*^2^ = 0.71). Post-hoc analysis indicated that the perceived pain intensity and unpleasantness ratings at time points in the range of 15–300 s were significantly higher than those at 480 s (i.e., the end of recovery phase) (all *Ps* < 0.05).

#### ERPs elicited by standard and deviant tones

ERPs generated in response to standard and deviant auditory stimuli were compared during the baseline, immersion, and recovery phases. Grand average waveforms recorded at Cz, Fz, and M2 are shown in Fig. 4B. N1 and P2 peak amplitudes and latencies at Cz are presented in Table 3. We observed a main effect of stimulus type on both N1 amplitude (F_1,15_ = 15.27, *P* = 0.001, *ηp*^2^ = 0.51) and P2 amplitude (F_1,15_ = 15.48, *P* = 0.001, *ηp*^2^ = 0.51), with both being larger in deviant trials than in standard trials. There was a main effect of phase (baseline, pain, recovery) (F_2,30_ = 6.70, *P* = 0.007, *ηp*^2^ = 0.31) on N1 peak latency and a main effect of stimulus type on P2 peak latency (F_1,15_ = 21.93, *P* < 0.001, *ηp*^2^ = 0.59). No other effects were found.

**Table 3:** Peak amplitude and latency of N1 and P2 to standard and deviant tones in cold pressor test at Cz electrode in experiment 2.

	Amplitude (μV)			Latency (ms)		
	Baseline	Pain	Recovery	Baseline	Pain	Recovery
***Standard***						
*N1*	−1.33 (1.32)	−1.42 (1.42)	−1.07 (1.09)	128.52 (18.87)	125.10 (24.16)	135.84 (10.27)
*P2*	2.60 (1.03)	2.53 (1.40)	2.37 (1.01)	213.97 (23.48)	213.23 (22.38)	204.20 (12.42)
***Deviant***						
*N1*	−2.94 (2.05)	−1.88 (1.87)	−2.23 (1.86)	130.47 (24.37)	133.15 (14.39)	151.47 (18.43)
*P2*	4.41 (3.18)	5.03 (3.08)	3.91 (2.26)	231.30 (46.77)	234.47 (20.71)	236.67 (25.40)

#### MMN

MMN waveforms corresponding to recordings made before (baseline), during (immersion), and after (recovery) the CPT at Fz and M2 are shown in Fig. 4C electrodes. Note that the MMN had a negative deflection at Fz and a positive deflection at M2.

**Figure 5:**
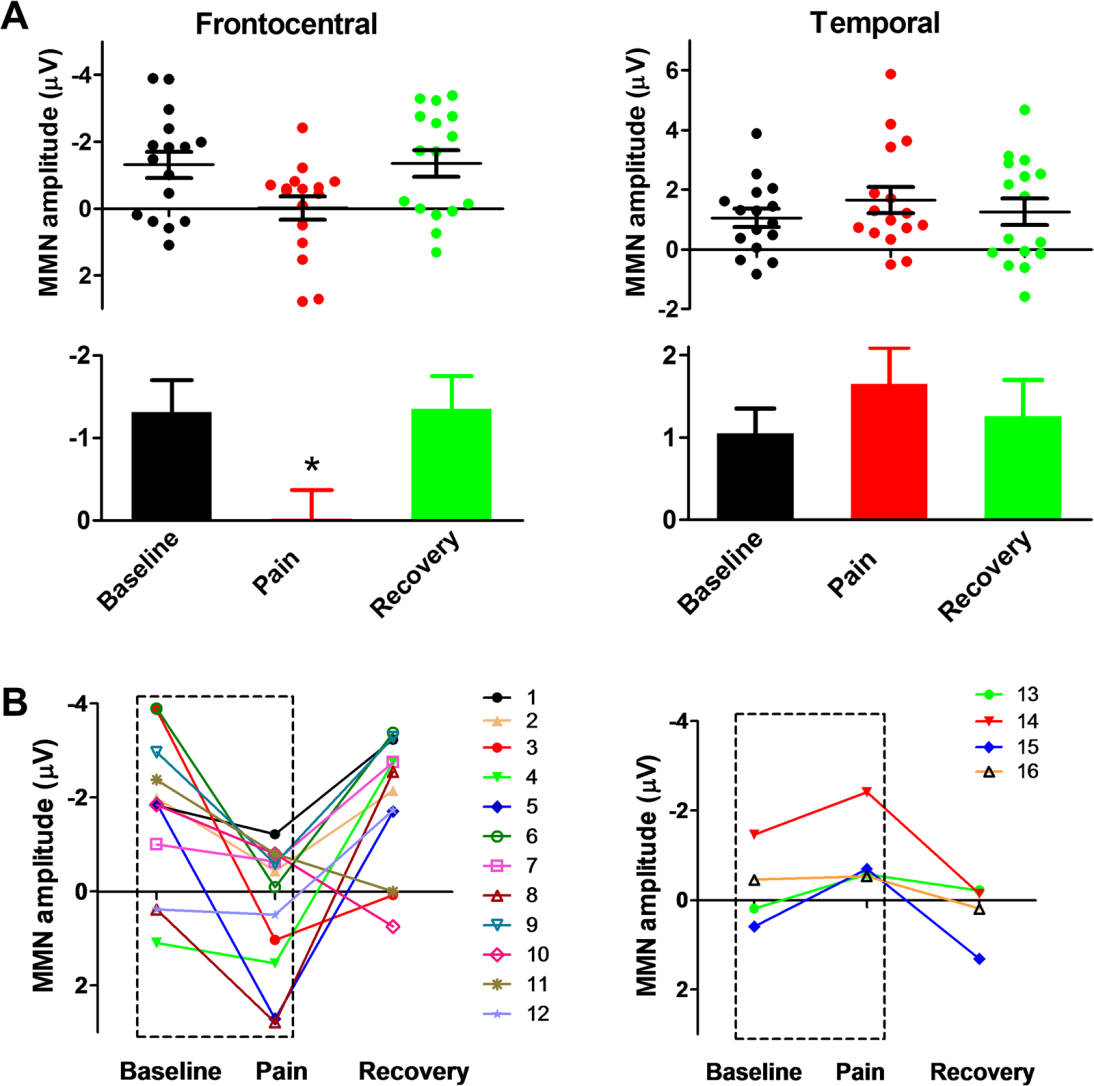
Comparisons of MMN amplitude between Experiment 2 phases and among individuals. (**A**) Reduced MMN amplitude in the ice-water immersion (pain) phase compared to that in the baseline and recovery phases. *P < *0*.*05*. (**B**) Different-colored numbers indicate different participants. Note the different response patterns observed.

Because a phase (baseline, immersion, recovery) × hemisphere two-way ANOVA did not detect a significant interaction, data were collapsed across the hemispheres. One-way ANOVAs across the three phases at frontocentral areas and at temporal areas (Fig. 5A) revealed a main effect of phase for the frontocentral MMN (F_2,30_ = 4.05, *P* = 0.031, *ηp*^2^ = 0.21). Post-hoc tests revealed that the MMN amplitude was reduced during ice-water immersion compared to that during the baseline (*P* = 0.038) and recovery phases (*P* = 0.032). No effect was found for the temporal MMN.

Changes in MMN amplitudes in frontocentral areas before, during, and after cold-water immersion for individual subjects are shown in Fig. 5B. MMN attenuation (towards positive deflection) due to cold-induced pain was observed in 12 of 16 subjects (75%). In the remaining 4 subjects, the reverse pattern occurred, with the MMN amplitude increasing (more negative deflection) in response to cold pain. No correlations were found between MMN amplitude and self-reported pain ratings.

## Discussion

The present study represents a first attempt to determine how pain affects auditory sensory memory in chronic and acute pain paradigms. Experiment 1 showed that TN patients had a smaller frontocentral MMN than the HCs with a right hemisphere dominance. This effect was observed in both nose- and mastoid re-referenced data analysis. When the ISI was increased, temporal MMN amplitudes decreased more rapidly in TN patients than in HCs, consistent with our hypothesis predicting that chronic pain patients would exhibit greater MMN attenuation in response to extending the ISI than HCs. Similar results were obtained in the mastoid-re-referenced data, corroborating the notion that chronic pain patients have impaired auditory sensory memory. Experiment 2 showed that MMN amplitude was reduced by acute cold pain and recovered after the pain was relieved, as we had hypothesized.

We observed a right hemisphere dominance of the MMN in both TN patients and HCs, consistent with previous studies showing a predominant right hemisphere scalp distribution of the MMN^37–39^. Likewise, a recent auditory evoked magnetic field study conducted in fibromyalgia patients showed that the MMN magnetoencephalographic equivalent has a right-hemisphere dominance lateralization pattern in response to tone stimuli^10^. This consistent laterality suggests that some MMN generation process is stronger in the right hemisphere than in the left hemisphere. Moreover, there appears to be a predominant right hemisphere frontal process associated with involuntary attention switching in response to an auditory change^11^.

It is noteworthy that our TN patients had a smaller frontocentral MMN than the HCs. This effect was observed in both nose- and mastoid re-referenced data analyzed across ISI conditions. These results are in agreement with Dick *et al*.’s findings of an increased MMN amplitude following analgesic treatment in chronic pain patients^12^. This pattern of findings may indicate a pain-related deficit in the initial encoding of sound stimulus properties. According to Eccleston *et al*.’s model, chronic pain, which is behaviorally distracting and disruptive, may in fact disrupt cognitive neurophysiological processes at very early processing stages associated with the MMN^40^. Empirically demonstrated attentional deficits in chronic pain patients, particularly in attention switching and attention interference tasks^41,42^, may be due to pain competing with other attention-demanding stimuli for limited cognitive resources^40,43^. Thus, chronic pain-induced reduction of the MMN may reflect disruption of pre-attentive processing.

The present data also provided evidence that the lifetime of a memory trace was reduced faster in chronic pain patients than in HCs. Both mastoid re-referenced frontocentral data and nose-re-referenced temporal data indicated that MMN amplitude decreased as a function of ISI in TN patients but not HCs. Generally, the MMN is thought to reflect an automatic change detection mechanism that is supported primarily by the perception of an incoming stimulus and the maintenance of its trace in memory^44^. According to Bartha-Doering *et al*., the duration and decay of auditory sensory memory can be examined by increasing ISIs, such that a prolongation of the ISI decreases MMN amplitude^45–47^. Consistent with this view, the significant main effect of ISI in the present study can be interpreted as a fading of the memory trace and the mastoid data are indicative of a hastened trace decay in chronic pain patients. This decreased MMN with a longer ISI may reflect a specific deficit in auditory sensory information maintenance and an impairment of echoic memory early in the progression of TN. Indeed, pain can slow stimulus information processing and impede working memory function^48^. Reports of poor memory and concentration among chronic pain patients are well known^5,49^.

In the present study, neither chronic pain nor acute induced pain affected temporal MMN, although there was a near-significant increase in the chronic pain condition for the short ISI. This finding may seem inconsistent with the main finding. We speculated that the increased temporal MMN in TN patients might be partially explained by a heightened vigilance (i.e., increased attention) to external stimulation in those suffering from chronic pain. The hypervigilance may result in a general enhancement in processing of sensory perception, including auditory discrimination, reflected as a somewhat larger temporal MMN.

Our finding of a significant correlation between MPQ sensory subscale score and MMN amplitude reduction across ISI conditions (long minus short) further confirmed that the greater the perceived intensity of pain, the faster auditory sensory memories decay. It was the sensory, but not the affective, component of pain that was associated with the sensory memory decay in chronic pain patients. It has been suggested that this MMN reduction results mainly from pain-related emotional disturbances, such as anxiety, distress, or depression^50,51^. Following Melzack *et al*.’s model of sensory, motivational, and cognitive determinants of pain^52^, the affective-motivational dimension of pain has been brought clearly into focus by clinical pain studies. Psychological factors have been shown to have a selective influence on the affective, versus the sensory, dimension of pain^53,54^. Nonetheless, the affective component of pain often covaries with pain intensity, a key feature of the sensory component of pain^55,56^. Pain intensity and unpleasantness ratings have often been reported to correlate strongly with each other^57,58^; the anterior cingulate cortex and medial thalamic nuclei, implicated in the encoding of the affective dimension of pain^59–61^, have also been found to exhibit pain intensity-related activation^55,62^.

The present study was the first to demonstrate that experimentally induced acute pain can disrupt the MMN. In contrast to Dick *et al*., who found that experimentally induced pain did not disrupt generation of the MMN^11^, we found that MMN amplitude was reduced by cold pressor pain and recovered after the pain was relieved. This inconsistency may be due to the different models used in the two studies, ischemic pain versus cold pressor pain. The former is thought to be caused by accumulation of algesic metabolites, occlusion of blood vessels below the inflated cuff, and mechanical pressure of the cuff, which activates mechano-sensitive nociceptors directly^63^. This physiological mechanism differs from that of cold-induced pain wherein direct activation of high-threshold thermo-sensitive nociceptors produces pain^64^. Another possible explanation for the discrepancy between findings is that the present study used an easy-to-detect tone differential (1000 Hz vs. 2000 Hz) while Dick *et al*.^11^ used both a difficult-to-detect (1000 Hz vs. 1020 Hz) and an easy-to-detect (1000 Hz vs. 1500 Hz) distinction. According to Sams *et al*., a greater standard-deviant distinction causes a larger MMN^65^. Thus, when the MMN is used as an index of cognitive impairment, the stimulus parameters used should be considered to ensure better sensitivity.

In conclusion, the current study represents a first attempt to determine how pain affects auditory sensory memory in chronic and acute pain conditions. We found that the auditory memory trace decayed faster in chronic pain patients than in HCs, and that experimentally-induced cold pain impaired pre-attentive auditory processing. Thus, we provided evidence that both trait pain and state pain can interfere with automatic change detection processes. Our study indicates that the MMN has the potential to be used as an objective index of clinical pain and therapeutic response. In future studies, it will be important to set up specific MMN recording parameters for different pain disorder etiologies.

## Data Availability

The datasets generated during and/or analyzed during the current study are available from the corresponding author on reasonable request.
